# Pediatric Project ECHO^®^ for Pain: implementation and mixed methods evaluation of a virtual medical education program to support interprofessional pain management in children and youth

**DOI:** 10.1186/s12909-023-04023-8

**Published:** 2023-01-28

**Authors:** C. Lalloo, V. Mohabir, F. Campbell, N. Sun, S. Klein, J. Tyrrell, G. Mesaroli, S. Ataollahi-Eshqoor, J. Osei-Twum, J. Stinson

**Affiliations:** 1 Child Health Evaluative Sciences, Peter Gilgan Centre for Research and Learning, The Hospital for Sick Children, 686 Bay Street, Toronto, ON M5G 0A4 Canada; 2 Institute of Health Policy, Management & Evaluation, University of Toronto, Toronto, ON Canada; 3 Department of Anesthesia and Pain Medicine, The Hospital for Sick Children, 555 University Avenue, Toronto, ON M5G 1X8 Canada; 4 Department of Anesthesiology and Pain Medicine, University of Toronto, 155 College Street, Toronto, ON M5T 1P8 Canada; 5 Department of Rehabilitation, The Hospital for Sick Children, 555 University Avenue, Toronto, ON M5G 1X8 Canada; 6 Department of Physical Therapy, University of Toronto, 155 College Street, Toronto, ON M5T 1P8 Canada; 7 Lawrence S. Bloomberg Faculty of Nursing, University of Toronto, 155 College Street, Toronto, ON M5T 1P8 Canada

**Keywords:** Pediatric pain, Project ECHO, Tele-education, Distance education, Continuing professional development, Mentorship, Community of Practice, Interprofessional

## Abstract

**Background:**

Pediatric pain is a complex health challenge requiring a multi-modal management approach. It is critical that healthcare providers (HCPs) have access to ongoing, flexible education and mentorship specific to pediatric pain. However, there are significant gaps in available pain education and a need for more opportunities to support interprofessional training. *Project Extension for Community Healthcare Outcomes (Project ECHO®)* is a model for delivering online HCP education and cultivating a virtual community of practice. Within the pediatric pain setting, *ECHO®* has potential to improve local access to specialized pain knowledge, particularly among the physicians, nurses, and allied health providers who primarily manage these cases in community and hospital settings across rural and urban environments. The purpose of this study was three-fold. First, to evaluate the feasibility (participation levels, acceptability) of implementing *Project ECHO®* in the context of pediatric pain. Second, to measure preliminary program impacts on HCP knowledge, self-efficacy, and clinical practice. Third, to characterize HCP program engagement levels before and after onset of the COVID-19 pandemic.

**Methods:**

A needs assessment was conducted to identify interprofessional education gaps and inform the program curriculum. The no-cost *Pediatric ECHO® for Pain* program offered TeleECHO sessions (didactic and case-based learning) as well as foundational education. Surveys were distributed at baseline and 6 months to assess outcomes using 7-point Likert scales. Participant engagement was assessed for periods prior to and during the COVID-19 pandemic. Descriptive and inferential statistical analyses were conducted.

**Results:**

Eighty-five TeleECHO sessions were hosted, with a mean attendance of 34.1 ± 23.4 HCPs. Acceptability scores at 6 months (*n* = 33) ranged from 5.0 ± 1.4 to 6.5 ± 0.5. Participants reported statistically significant (*p* < 0.05) improvements in knowledge (7 out of 7 topics) and self-efficacy (8 out of 9 skills). Most participants reported positive practice impacts, including improved satisfaction with managing children with pain. Exploratory analyses showed a trend of greater engagement from *ECHO®* learners after onset of the COVID-19 pandemic.

**Conclusions:**

*Project ECHO®* is a feasible and impactful model for virtual education of interprofessional HCPs in managing pediatric pain.

**Supplementary Information:**

The online version contains supplementary material available at 10.1186/s12909-023-04023-8.

## Background

Pediatric pain is a significant health problem that can impair all aspects of life, including sleep, mood, physical functioning, peer and family relationships, and school attendance [1–4]. Pediatric acute pain commonly results from surgery, trauma (e.g. burns, motor vehicle accidents, sports injuries), diseases (e.g. sickle cell crises, juvenile idiopathic arthritis), and procedures (e.g. vaccinations, blood draws) [5]. Examples of chronic pain in children include headache, abdominal pain, musculoskeletal pain, and neuropathic pain [6]. Chronic pain impacts 1 in 5 children and youth in Canada, and disproportionally affects Black, Indigenous, and other racialized individuals [7]. Early pain management intervention is critical to reducing pain-related disability, duration, and healthcare costs [3, 4]. However, wait times to access specialized pain treatment can often extend for months or even years [8, 9]. Therefore, as recommended by the Canadian Pain Task Force, it is pivotal to engage health professionals from primary and secondary care settings in the management of pediatric pain [7, 10].

The optimal treatment model for pediatric pain is an interdisciplinary “3-Ps” approach combining pharmacological, physical, and psychological strategies [11]. In general, the most severe and highly impaired pediatric cases are managed by specialized pain clinics (e.g. post-operative chronic pain, trigeminal neuralgia, sickle cell crises) while the majority are managed by community healthcare providers (HCPs) [9, 12]. Balancing multiple symptoms and needs in the community can often lead to challenges related to treatment fragmentation and uncertainty around patient pathways to accessing pain care [10]. Indeed, a 2019 national survey identified “better access to pain care” as a top priority of Canadian families affected by pediatric pain [13].

The COVID-19 global pandemic necessitated a well-documented shift away from in-person pain care in favour of virtual care options [14, 15]. A less-documented corollary is the need to transform traditional in-person interprofessional health education for remote delivery of educational activities, while optimizing learner outcomes. Moreover, the rapidly changing healthcare landscape makes it critical that HCPs have access to ongoing, flexible education and mentorship to support local management of pediatric pain [16]. However, according to a recent report from the Canadian Pain Task Force, there are significant gaps in available pain education for HCPs and a need for more opportunities to support interprofessional training in pain management [10]. This pain education gap does not only impact Canadian HCPs. Indeed, this issue impacts specialty medical education for HCPs across North America and globally with the World Health Organization recommending that all countries prioritize pain management through education to build global capacity to improve management worldwide [17, 18].

*Project ECHO®* (Extension for Community Healthcare Outcomes) is a model for technology-enabled interprofessional education and development of a virtual community of practice [19, 20]. It is designed to expand access and capacity to deliver evidence-informed specialized care in local settings. Specifically, the *ECHO®* model provides HCPs with training, mentorship, and support to locally manage their patients with specialized health needs. *ECHO®* uses a “Hub-and-Spoke” structure, wherein the Hub (a specialty interprofessional team) regularly connects via videoconference with multiple Spokes (community-based HCPs) to learn together with the shared goal of enhancing local patient care.

The *ECHO®* model has been successfully applied across a wide range of clinical conditions in adults (e.g. chronic pain, opioid stewardship, concussion) and pediatrics (e.g. palliative care, complex care) [21–25]. Within the pediatric pain setting, *ECHO®* has potential to improve local access to specialized pain knowledge, particularly among the physicians, nurses, and allied health providers who primarily manage children with pain in community and hospital settings across rural and urban environments. However, while approximately 10% of all *ECHO®* programs worldwide concentrate on pediatric healthcare, none focus on acute and chronic pain management in children [26].

Prior to onset of the COVID-19 pandemic, with support from Canada’s largest provincial Ministry of Health (MOH), our group sought to adapt the *ECHO®* model to focus on managing acute and chronic pain in children and adolescents. While the program was designed for HCPs practicing in Ontario, it has since generated national and international participation through word-of-mouth. This paper will describe the process of developing, implementing, and evaluating *Pediatric Project ECHO® for Management of Pain in Children and Youth (Pediatric ECHO® for Pain)*. It will also consider the suitability of the *ECHO®* model to facilitate engaging interprofessional health education during public health emergencies, such as the COVID-19 pandemic.

## Methods

### Objectives

The primary objective of this research study was to determine whether it was feasible to implement the *Pediatric ECHO® for Pain* program based on participation and program acceptability. The secondary objective was to characterize perceived impacts of *Pediatric ECHO® for Pain* on participant knowledge, self-efficacy, and clinical practice after 6 months using a pre/post study design. The exploratory objective was to characterize attendance and engagement levels with *Pediatric ECHO® for Pain* before and immediately after onset of the COVID-19 pandemic.

### Needs assessment to inform curriculum development

A needs assessment was conducted prior to program launch to inform development of a pediatric pain-specific *Project ECHO*® curriculum [27]. An online survey (49 items; 15-minutes) was distributed via targeted emails to professional networks, medical and nursing associations, and allied health organizations throughout Canada. Administered using Research Electronic Data Capture (REDCap), the survey was designed to assess interest in specific topics using individual Likert scales ranging from 1 (“no interest”) to 5 (“very interested”). The list of topics was developed through clinician consensus (including authors FC, JT, GM). Survey respondents could also suggest additional topics of interest through open-text fields. See Supplemental Materials for survey items and curriculum topics (Additional file 1). The survey was live between May and August 2017.

### Recruitment of spokes

For initial program implementation, resources focused on leveraging the expertise of healthcare professionals working in the five provincial interprofessional Chronic Pain programs, all part of the Ontario Chronic Pain Network. Marketing materials were disseminated through this network with the aim of recruiting HCPs to the program. Additionally, materials were disseminated broadly through pediatric pain-focused conferences, organizations, and mailing lists. *Pediatric ECHO® for Pain* launched in October 2017. While registration for *Pediatric ECHO® for Pain* was targeted at interprofessional HCPs in Ontario, any HCP or individual with an interest in pediatric pain could register as a participant at no cost and earn continuing professional development credits. These credits were sent to individuals who registered and attended a session. HCPs could join at any time (i.e., rolling recruitment). After registering for the program, participants received regular email notifications of upcoming ECHO® sessions. This program has demonstrated global reach with HCPs from over 14 unique low-middle-income countries in attendance (e.g. Nigeria, India, Kenya, Mexico, Bangladesh, South Africa).

### Program structure

**Fig. 1:**
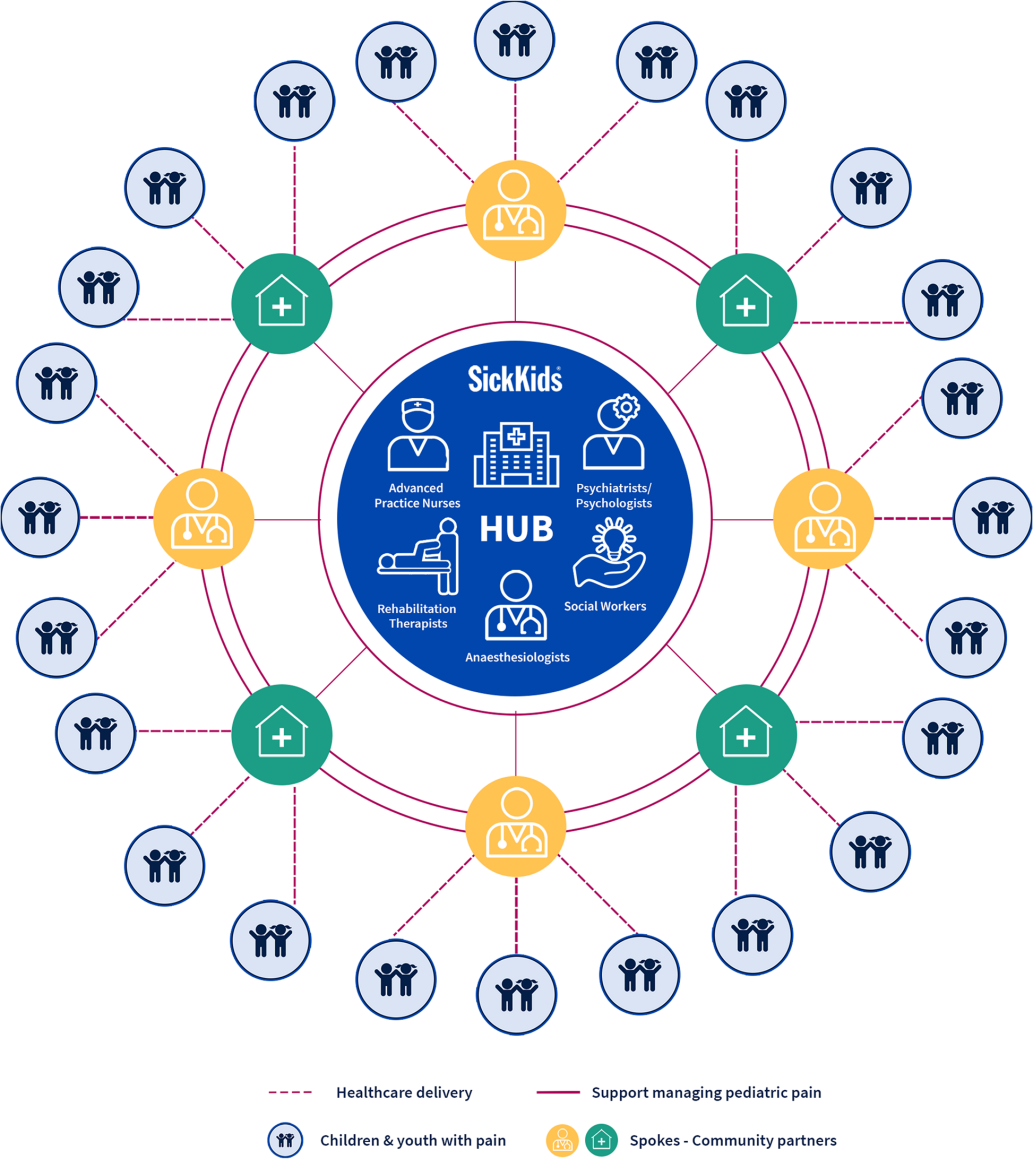
Structure of *Pediatric ECHO® for Pain*, a technology-enabled virtual education program to support interprofessional management of children with acute and chronic pain

*Pediatric ECHO® for Pain* is based at The Hospital for Sick Children (SickKids) in Toronto, which is the largest pediatric tertiary care hospital in Canada. The program is funded by the Ontario Ministry of Health, initially as a demonstration project (2017–2019), and subsequently through annual operational funding (2020 onward). The demonstration period involved evaluating program feasibility from the perspective of the operations team, HCP learners, and hub team for *Pediatric Project ECHO for Pain*. The operational period is characterized by ongoing support from the MOH to deliver the program free-of-charge to HCPs. Day-to-day program logistics are overseen by an Operations team while program delivery is facilitated by clinicians from the SickKids Pain Program in the role of ECHO leads (including authors FC, JT, NS, GM, SAE, SK) and interprofessional members of the wider hub team. The SickKids Pain Program cares for children from across Ontario, including those residing in remote and rural regions including Northern Ontario. The team of specialists at the *Project ECHO®* academic Hub has included representatives from advanced practice nursing, anesthesiology, physiotherapy, occupational therapy, psychology, and social work (see Fig. 1). Program evaluation is overseen by an independent research team (authors CL, VM, JOT, JS).

*Pediatric ECHO® for Pain* offers virtual interactive seminars called TeleECHO clinics (60 minutes). Each TeleECHO clinic includes a didactic presentation (~ 15 minutes), a de-identified case presentation by a community HCP (~ 20 minutes), and facilitated discussion to generate evidence-informed recommendations for managing the presented case (~ 25 minutes). TeleECHO clinics were offered biweekly during the demonstration period (i.e., total of 23 sessions), and weekly during the operational period (i.e., 12 sessions over 12 weeks). TeleECHO clinics are conducted virtually using Zoom videoconferencing technology with support from the telemedicine department at SickKids. In addition to TeleECHO clinics, the program also offered a distinct Core Competency curriculum focused on pediatric pain. The Core Competency was delivered over Zoom in 8 weekly installments (60 minutes each). Individuals with an interest in pediatric pain could register separately for the TeleECHO clinics and/or the Core Competency curriculum.

### Program evaluation

Each *Pediatric ECHO® for Pain* program participant was invited to take part in an embedded research study at the time of registering for the program. All procedures performed in studies involving human participants were in accordance with the ethical standards of the Hospital for Sick Children Research and Ethics Board (Approval #1000057321), the Helsinki Declaration and its later amendments, and the Canadian Tri-Council Policy Statement on Ethical Conduct for Research Involving Humans (TCPS-2). Informed consent was obtained from all participants in the study online via REDCap, a secure web-based application hosted at SickKids. Surveys were distributed to consented participants online using REDCap [28]. Surveys were reviewed by interprofessional education specialists and hub team members. Participation levels at each TeleECHO clinic and Core Competency session were centrally tracked and electronic surveys were distributed at the time of registration (baseline). Separate surveys were distributed for each program component (i.e., TeleECHO clinic, Core Competency). Participants who attended at least one TeleECHO clinic in the 6 months following registration were eligible to complete the corresponding follow-up survey (i.e., 6 months after baseline). Participants who attended at least one Core Competency session during the 8-week curriculum were eligible to complete the corresponding follow-up survey (i.e., 8 weeks after baseline). Surveys were designed to assess program acceptability as well as perceived knowledge and self-efficacy for topics and skills from the program. Survey items were developed to accommodate the interprofessional nature of program participants, as no existing validated knowledge or self-efficacy questionnaires related to the management of pediatric pain were suitable for all health professionals. Consequently, the *Pediatric ECHO® for Pain* surveys were purposefully designed such that respondents could align their self-assessments of knowledge and self-efficacy with best judgement of their own scope of practice. Knowledge and self-efficacy items were framed with the stem, *“for my scope of practice, I currently have an appropriate level of knowledge about [topic]”* and *“for my scope of practice, I am confident in my ability to [skill]”*. Likert scales were used to assess item-level agreement, where 1 indicated “strongly disagree” and 7 indicated “strongly agree”. “Not applicable” options were provided when relevant. Global changes in knowledge and practice were assessed upon completion of the Core Competency curriculum via a one-item scale based on the Patient Global Impression of Change measure [29]. Survey items assessing perceived clinical practice impact were derived from *ECHO*® literature and research/clinical expertise of the program team. Participant reflections on the program were also collected through open-text responses on the follow-up survey. Participants were offered a token of appreciation ($5 electronic gift card) upon completion of their 6-month survey.

### Data analysis

Quantitative data were exported from REDCap and analyzed using Stata Basic Edition Version 17 [30]. To protect the confidentiality of participants, demographic data on professional role were collapsed into an “Other” category for items with only one respondent. Qualitative data from open text fields were exported verbatim from REDCap and organized by authors CL, JOT, and VM. Curriculum preferences from the needs assessment survey were sorted descriptively. The a priori target number of attendees at each TeleECHO clinic and Core Competency session was > 6, based on previous pilot *ECHO®* programs [21]. The target threshold for acceptability was a mean score of > 5 (7-point Likert scale) across all survey items. No thresholds were specified for perceived knowledge, self-efficacy, or practice impact. Knowledge items were collapsed into categories based on clinical area by authors CL, VM, JOT, NS, GM, and SK. For TeleECHO clinics, paired sample t-tests were used to examine changes in knowledge and self-efficacy items from baseline to 6 months among those participants who completed both surveys. In order to characterize participant engagement before and during the COVID-19 pandemic, audio recordings of TeleECHO clinics were reviewed by author JOT. “Engagement” was operationally defined to include the following discrete interactions: asking or answering a question, making a comment, or posting in the virtual chat. All surveys with complete responses from program inception (2017) to the end of Cycle 6 (March 2022) were included in the analysis. A sub-analysis was completed for a comparative analysis of attendance and engagement during pre-pandemic (November 2019 to February 2020) and post-pandemic (September to November 2020) periods.

## Results

### Needs assessment

Respondents were interprofessional with a total of 35 respondents from 21 unique healthcare institutions. They included: Nurses (*n* = 12), Physicians (*n* = 10), Dietitians (*n* = 4), Rehabilitation Therapists (*n* = 3), Psychologists (*n* = 2), Social Workers (*n* = 2), Pharmacist (*n* = 1), and a Program Manager (*n* = 1). Topics of interest spanned the interdisciplinary “3-Ps” approach to pediatric pain management including physical (e.g., desensitization), psychological (e.g., mind-body techniques) and pharmacological (e.g. medications for neuropathic pain) strategies as well as educational resources for children and families (e.g. explaining pain mechanisms). Service delivery was also highlighted as an education need by participants (e.g., supporting transition to adult care). These educational preferences were used to inform the curriculum for *Pediatric ECHO® for Pain*. For instance, the identified need for education on ‘desensitization’ was addressed through sessions focused on advanced physiotherapy modalities for pediatric chronic pain. Given that the quantity of suggested topics exceeded the number of *ECHO®* sessions, topics were integrated into the initial curriculum based on respondent interest and speaker availability. The curriculum was accredited through the University of Toronto such that participants could earn continuing professional development credits.

### Program feasibility

During the study period (April 2017 to March 2022), 85 TeleECHO clinics and 2 cycles of Core Competency (November 2017 to January 2019) were completed. The TeleECHO clinics included a mean (SD) of 34 attendees (±23.4). Attendance varied each month, from a minimum of 6 to maximum of 138 participants. A total of 37 individuals registered for the Core Competency and these sessions had an average (SD) attendance of 13 (±3.70). The characteristics of program attendees who completed follow-up surveys are described in Table 1. Overall, participants included representation from all 14 (100%) Local Health Integration Networks, which are sub-regions spanning Ontario that were responsible for health administration from 2006 until their dissolution in 2019 [31].

**Table 1:** Characteristics of *Pediatric ECHO for Pain* participants

	Program registrants with completed baseline survey who also attended ≥ 1 session, ***n*** = 105	
Characteristic	Did not complete follow-up survey ***n*** = 72	Completed follow-up survey ***n*** = 33
Sex, n (%)		
Female	59 (81.9)	28 (84.8)
Male	7 (9.7)	4 (12.1)
Other	2 (2.8)	0 (0.0)
Missing data	4 (5.6)	1 (3.0)
Age Group, n (%)		
≤ 29 years	15 (20.8)	3 (9.1)
30–49 years	39 (54.2)	21 (63.6)
50–69 years	15 (20.8)	8 (24.2)
Missing data	3 (4.2)	1 (3.0)
Profession, n (%)		
Nurse^a^	29 (40.3)	10 (30.3)
Pharmacist	4 (5.6)	0 (0)
Physician^b^	8 (11.1)	4 (12.1)
Psychologist	5 (6.9)	1 (3.1)
Rehabilitation Therapist^c^	16 (22.2)	4 (12.1)
Other^d^	10 (13.9)	13 (39.4)
Missing data	0 (0.0)	1 (3.0)
Primary Practice Setting, n (%)		
Academic hospital	42 (58.3)	10 (30.3)
Community Health Centre (CHC)	6 (8.3)	3 (9.1)
Non-academic hospital	4 (5.6)	4 (12.1)
Private practice	2 (2.8)	7 (21.2)
Other^e^	16 (22.2)	7 (21.2)
Missing data	2 (2.8)	2 (6.1)
Years in Practice, n (%)		
Less than 1 year	7 (9.7)	3 (9.1)
1–4 years	15 (20.8)	2 (6.1)
5–10 years	15 (20.8)	8 (24.2)
Greater than 10 years	32 (44.4)	18 (54.5)
Not applicable	2 (2.8)	1 (3.0)
Missing data	1 (1.4)	1 (3.0)

^a^Nurse – Registered Practical Nurse, Registered Nurse, Nurse Practitioner, Clinical Nurse Specialist; ^b^Physician – Pediatric Resident, Primary Physician, Physician (specialist); ^c^Rehabilitation Therapist – Kinesiologist, Occupational Therapist, Physical Therapist; ^d^Other – Chaplin, Child Life Specialist, Data Analyst, Dentist, Physician Assistant, Psychological Associate, Registered Massage Therapist, and Social Worker; ^e^Other – Hospice, Education, Home Care

### Program acceptability

Participant perceptions of acceptability with the TeleECHO clinics and Core Competency program components are presented in Table 2. In addition, 2 TeleECHO participants provided written feedback on program acceptability via open-text fields on the 6-month survey. This feedback supported the generally high ratings of program acceptability.

- For instance, a Rehabilitation Therapist participant stated, *“[I] learned there are no borders to cross if one wants to have education. Our present technology gives us the opportunity to be global, national, local, remote providers and learners.”*
- A Psychologist participant shared that the program provided, *“confirmation that [my] current approach is consistent with practice at other centres.”*

**Table 2:** Acceptability and satisfaction with program components. Possible item scores ranged from 1 to 7 where 1 = ‘strongly disagree’ and 7 = ‘strongly agree’. “NA” indicates that corresponding item was not part of the Core Competency follow-up survey

	TeleECHO Clinics, 6 Months, ***n*** = 33			Core Competency, Post-Program, ***n*** = 11		
Item	Mean	SD	Min, Max	Mean	SD	Min, Max
Involvement in the program is a worthwhile experience for me.	6.2	1.1	2, 7	6.2	0.9	5, 7
The program is an effective way for me to learn.	5.8	1.4	2, 7	5.8	1.1	4, 7
The program has created a supportive community of practice.	5.0	1.4	1, 7	5.6	1.3	3, 7
I would recommend the program to my colleagues.	5.5	1.2	2, 7	*NA*	*NA*	*NA*
The program has connected me with peers and diminished my professional isolation.	6.0	1.0	3, 7	*NA*	*NA*	*NA*
I have learned new information.	6.1	1.1	2, 7	*NA*	*NA*	*NA*
I have learned best practice care.	5.8	1.4	2, 7	*NA*	*NA*	*NA*
I respect the knowledge of the facilitators.	6.5	0.5	6, 7	*NA*	*NA*	*NA*

### Knowledge of pediatric pain

Among TeleECHO clinic participants (*n* = 33), there were significant improvements in topic-specific knowledge scores across all categories from baseline to 6 months (see Table 3). Among Core Competency participants (*n* = 11), 90% reported an improvement in global knowledge related to managing pediatric pain and 36% indicated that this improved knowledge had translated into practice change. Five Core Competency participants provided a rationale for their reported global knowledge change score. Participants who reported their knowledge change as “a little better” shared:

- “*I do not have the opportunity to apply the knowledge to children. I have been using it for adults though!*” (Nurse Practitioner)
- “*[I provide] limited direct patient care.*” (Clinical Nurse Specialist)

**Table 3:** Knowledge scores among TeleECHO participants of *Pediatric ECHO for Pain*, at baseline and 6 months. Possible item scores ranged from 1 to 7, where 1 = ‘strongly disagree’ and 7 = ‘strongly agree’. ‘3Ps’ refers to the multimodal management of pain (pharmacotherapy, physical, psychological)

Knowledge Category (***N*** = 33)	Baseline (SD)	6 Months (SD)	Mean Difference	Paired t-test	
				***P***-value	95% CI
Assessment (physical, mental)	3.7 (1.2)	4.9 (1.0)	−1.2	0.000	−1.8 – −0.7
Sleep assessment and management	3.8 (1.7)	5.4 (1.2)	−1.6	0.000	−2.4 – − 0.9
Managing chronic pain	4.4 (1.0)	5.3 (1.0)	-0.9	0.001	− 1.4 – − 0.4
Populations with special considerations	3.6 (1.2)	4.9 (1.1)	−1.3	0.000	−1.9 – −0.7
Pharmacotherapy	3.1 (1.5)	4.6 (1.6)	−1.5	0.000	−2.2 – −0.7
Physical strategies	4.7 (1.9)	6.5 (1.6)	−1.8	0.000	−2.7 – −1.0
Psychological strategies	5.7 (2.2)	7.3 (1.4)	−1.6	0.001	−2.8 – −0.7

Among those participants who reported “somewhat better” or “moderately better”

- “*[We are] still developing our pediatric palliative care practice.*” (Anaesthesiologist)
- “*I choose this rating as truly my participation has given me a better knowledge base although not significant changes in my practice although it has given me program ideas to discuss with my supervisor*”. (Rehabilitation Therapist)
- “*I haven’t had a lot of opportunity with my current clients to put into practice everything I’ve learned.*” (Physiotherapist)

### Self-efficacy

Among TeleECHO clinic participants, there were significant improvements in self-efficacy scores for the majority of assessed skills from baseline to 6 months (see Table 4). Among Core Competency participants (*n* = 11), 81% reported an improvement in global confidence in managing pediatric pain and 36% indicated that this improved self-efficacy had translated into practice change.

**Table 4:** Self-efficacy scores among TeleECHO participants of *Pediatric Project ECHO for Pain*, at baseline and six months. Possible item scores ranged from 1 to 7, 1 = ‘strongly disagree’ and 7 = ‘strongly agree’

Self-efficacy	n	Baseline (SD)	6 Months (SD)	Mean Difference	Paired t-test	
					***P***-value	95% CI
Manage children with chronic pain.	32	4.2 (1.5)	5.3 (1.7)	−1.2	0.004	−2.0 – −0.4
Assess pain in children.	33	4.5 (1.8)	5.7 (1.4)	−1.2	0.003	−2.0 – −0.4
Identify children with pain needs.	33	5.0 (1.3)	5.8 (1.4)	−0.8	0.012	−1.5 – − 0.2
Understand possible side effects of most pharmacological medications used for pain.	33	4.1 (2.0)	4.9 (1.9)	−0.8	0.102	−1.7 – 0.2
Understand appropriate mind-body/psychological interventions for children with pain.	32	4.3 (1.5)	5.4 (1.2)	−1.1	0.002	−1.8 - -0.5
Educate parents and caregivers about pain.	32	4.8 (1.5)	5.9 (1.2)	−1.1	0.001	−1.8 – −0.4
Serve as an expert in my community for pain-related questions and issues.	17	3.6 (1.8)	5.1 (1.9)	−1.5	0.029	−2.8 - -0.2
Communicate effectively with children and community members about pain.	33	4.5 (1.6)	5.5 (1.3)	−1.1	0.005	−1.8 - -0.3
Understand appropriate physical interventions for children with pain.	32	4.3 (1.4)	5.5 (1.2)	−1.2	0.000	−1.9 - -0.6

### Perceived impact on clinical practice

**Fig. 2:**
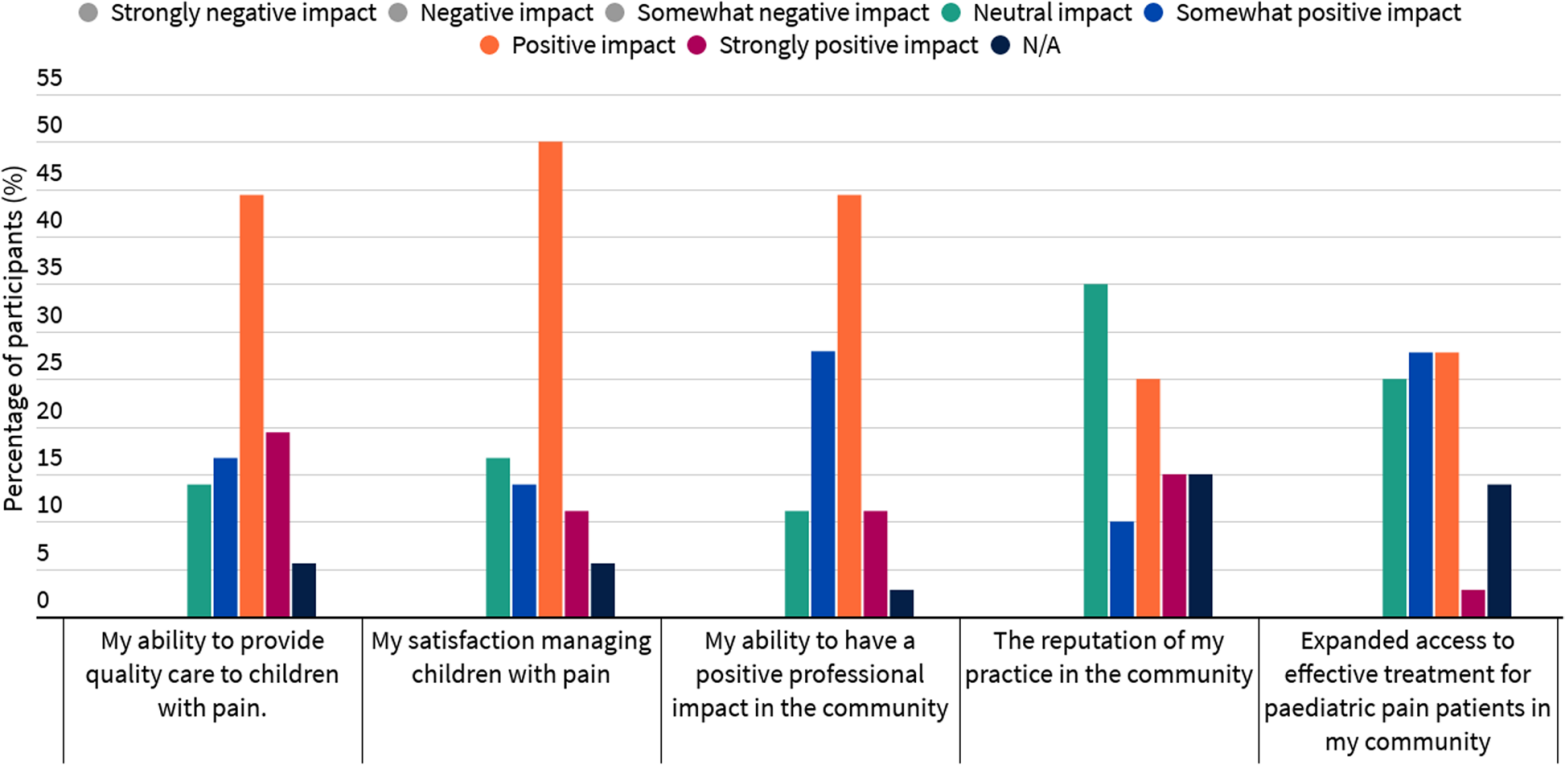
Practice impacts of *Pediatric ECHO® for Pain* after 6 months of participation, *n* = 36. Possible response options ranged from “strongly negative” to “strongly positive”. Only endorsed responses are displayed. Due to rounding, percentages may not total to 100%

The reported impact of TeleECHO participation on various aspects of clinical practice is presented in Fig. 2. A total of 34 participants provided written feedback on the survey items related to clinical practice. Those who reported a neutral or somewhat positive impact noted that:

- *“I kept my interaction brief.”* (‘Other’ HCP)
- *“Currently not seeing clients on a regular basis.”* (Rehabilitation Therapist)
- *“I’m also up for further education but it hasn’t changed the way I practice [with respect to] a consultative style of practice.”* (Rehabilitation Therapist)
- *“Rarely see pediatric patients with chronic pain, but good to know the information.”* (Nurse Practitioner)
- *“My knowledge is definitely better. However, in my practice … I am working on being able to translate some of this knowledge to work with potential clients.”* (Rehabilitation Therapist)

Those participants who reported a positive practice impact stated:

- *“Very practical information, able to treat and educate patients more effectively.”* (Rehabilitation Therapist)
- *“I was able to apply some of my learning.”* (Nurse Practitioner)
- *“Improvement in understanding the role of other disciplines in treatment of chronic pain.”* (Psychologist)
- *“One doesn’t learn everything at school. Until you work with people, you cannot learn. I got an opportunity to learn from different healthcare providers in different fields. And when I put all together, I finished the puzzle.”* (Rehabilitation Therapist)

Thirteen TeleECHO clinic participants out of 33 respondents (39%) indicated that program discussions changed the care plan for their patients. For those participants who had opportunities to implement new skills, they shared examples such as:

- *“I was able to look at my client from all available different angles in relation to assessment, treatment plan etc. I was able to modify some existing non effective behaviours into effective behaviours. I was able to learn the continuum of the learning behaviour.*” (Rehabilitation Therapist)
- *“Highlighted strategies to support students and identifying their health needs as related to pain and anxiety and the students barriers to success at school.”* (Rehabilitation Therapist)
- “*Follow up.*” (Psychologist)
- “*More treatment options.*” (Rehabilitation Therapist)
- “*Gave me a better basis for assessments/treatment.*” (‘Other’ HCP)

### Project ECHO engagement and the COVID-19 pandemic

**Fig. 3:**
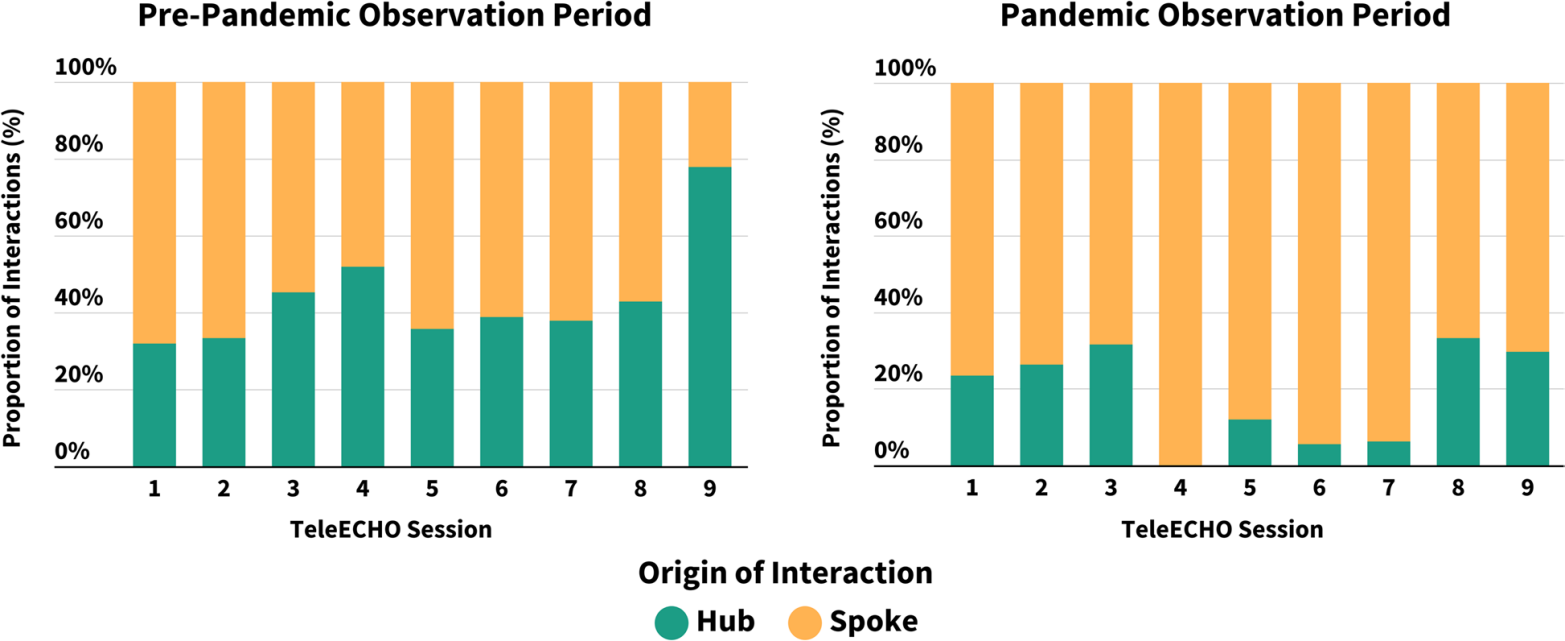
Participant engagement with *Pediatric ECHO® for Pain* during pre-pandemic and pandemic observation periods, *n* = 18. *One session was excluded from analysis of the pre-pandemic period because it did not include the typical didactic component. Similarly, one session was excluded from analysis of the pandemic period because it did not include the typical case presentation component

During the pre-pandemic observation period (37 sessions), an average of 21 HCPs (SD 10.2; range 6–52) attended TeleECHO clinics, compared to an average of 46 HCPs (SD 24.8; range 17–138) during the pandemic observation period (48 sessions). In terms of engagement, there were a similar number of TeleECHO interactions generated during the pre-pandemic (mean 26.7, SD 7.1) and pandemic (mean 25.6, SD 7.4) observation periods. However, the origin of these engagement interactions trended from being primarily Hub-driven to primarily Spoke HCP-driven, as visualized in Fig. 3.

## Discussion

This study sought to develop the first *ECHO®* program focused on pediatric pain, evaluate its feasibility and preliminary impact, and characterize its application before and during the COVID-19 pandemic. The *ECHO®* model, originally designed for physicians with an interest in hepatitis C, was purposefully adapted to accommodate interprofessional learners with an interest in pediatric pain from primary care, community hospital, and tertiary care practice settings. *Pediatric ECHO® for Pain* has a non-hierarchal structure and a focus on collaborative interprofessional learning. Results indicate that the program is feasible to implement, with participation levels over the first 2 years (2017–2019) averaging 17 participants per session. Scores of program acceptability also exceeded a priori thresholds for most assessed items (7/8; 87.5%). On average, participants reported significantly improved pediatric pain knowledge for all topics and enhanced self-efficacy for most skills related to pain management after 6 months. Most participants indicated that the program had a positive impact on various aspects of their practice, including their ability to provide quality care to children with acute or chronic pain needs. Comparative explorations of participant engagement in relation to the COVID-19 pandemic revealed a shift toward spoke-driven (i.e., community HCPs and those with an interest in pediatric pain) engagement during the pandemic observation period.

Consistent with previous *ECHO®* research, participants indicated that *Pediatric ECHO® for Pain* was an effective way to learn and that their voluntary program participation was worthwhile [21, 25, 32–36]. However, on average, participants reported a ‘neutral’ response (i.e. mean score of 4.6 on 7-point Likert scale) to the acceptability item, “*the program has connected me with peers and diminished my professional isolation*”. The heterogeneous nature of pediatric pain as a clinical problem, which intersects many different conditions and areas of practice, may influence the ability to forge a sense of professional connectedness. There may be a need for supplementary initiatives to diminish professional isolation of community spokes who care for children with pain. Our findings suggest that evaluating outcome measures following 6 months of participation in *Pediatric ECHO® for Pain* is sufficient to observe changes in knowledge categories regarding the assessment, management, and treatment of acute and chronic pain in pediatric populations and most accompanying skills. Furthermore, the improved knowledge and self-efficacy scores found in this study are comparable with results noted for adult chronic pain *ECHO®* programs and other *ECHO®* programs [20, 21, 35, 36]. By conducting a needs assessment prior to the launch of the program, we ensured that curriculum topics were relevant to HCPs with varying scopes of practice and those with an interest in pediatric pain. Knowledge and self-efficacy findings provide additional support for the implementation of *ECHO®* as an effective interprofessional education model to meet identified learning needs [10]. Content analysis of open-ended survey responses highlighted the importance of learning multiple strategies and techniques for pediatric pain management, and combining, comparing, and customizing pain management solutions which best fit the needs of an individual patient. Future studies should explore the qualities of an *ECHO* session which contribute to increased knowledge, self-efficacy, and positive practice impacts (e.g. presence of interprofessional learners) [37].

The COVID-19 pandemic provided a unique opportunity to explore the applicability of the *ECHO®* model for the remote delivery of education during public health emergencies. To our knowledge, this is the first *ECHO®* program to report on changes in participant engagement before and during the COVID-19 pandemic. While exploratory, the observed trends toward more spoke-driven engagement may reflect the increased availability of HCPs to attend TeleECHO sessions due to decreased clinical load, increased need for mentorship among community-based participants, an increased familiarity and comfort with technology due to ubiquitous adoption during the pandemic, and/or a strengthened community of practice due to the shared experience of providing healthcare during a pandemic. Future research will build upon these preliminary findings by examining longer-term trends and exploring perspectives of Hub and Spoke participants using qualitative methods.

### Strengths and limitations

The pre- and post-intervention study design with iterative program cycles, wherein participants served as their own control, was one strength of this study. Program participants represented numerous professions and clinical disciplines, which emulates care management of pediatric pain patients in North America and increases the generalizability of findings to a broad group of HCPs. Another study strength was the study surveys used to assess knowledge and self-efficacy, which were derived from needs assessment findings and were designed to account for interprofessional scopes of practice among participants. One limitation of this study was the modest response rate for the baseline and follow-up surveys. With the use of standardized, REDCap-generated email reminders, the baseline survey response rate was 45% (*n* = 167/369) and the 6-month response rate was 45% (*n* = 33/74). These response rates are consistent with a recent systematic review on the Project ECHO model [38]. Accordingly, selection bias may influence our findings, as a subset of participants may be more likely to attend TeleECHO clinics, Core Competency sessions and elect to complete surveys.

Future research should seek to investigate the impact of *Pediatric ECHO® for Pain* on patient-level outcomes. This research should analyze the implementation of concepts learned in *Pediatric ECHO® for Pain* using measures to supplement self-report (e.g. prescribing behaviour, referrals for multi-disciplinary HCPs). Such outcomes are challenging to assess, as the *ECHO®* Hub does not have direct contact with the patients who are managed by Spoke participants in the community. This research is still in its infancy and will require careful consideration of study design and appropriate measures of pediatric pain outcomes. Given that *Pediatric ECHO® for Pain* is the first such *ECHO®* program to focus on acute and chronic pediatric pain, additional research is also required to determine whether observed changes in knowledge and self-efficacy are sustained beyond the 6-month time point, and whether positive practice impacts are sustained with continued program participation. Qualitative methodology could also be utilized to learn more about the components of TeleECHO that are most effective for pediatric pain education.

The *ECHO®* model, originally developed at the University of New Mexico for the management of hepatitis C, was successfully implemented in Canada in response to gaps in care related to the management of acute and chronic pain in children. This study demonstrates the feasibility and acceptability of *Pediatric ECHO® for Pain* among community providers. The program was successful in achieving positive and significant changes in knowledge and self-efficacy, as well as moderate positive practice impacts. Implementation before and during the COVID-19 pandemic suggests that *ECHO®* offers an attractive model to facilitate engaging virtualized medical education amid public health emergencies.

In conclusion, the *ECHO®* model is a feasible and impactful approach to support interprofessional HCPs in caring for children living with acute and chronic pain during and beyond onset of the COVID-19 pandemic.

## Supplementary Information

**Additional file 1.** Curriculum summary of Pediatric Project ECHO for Pain (2017-2022).

## Data Availability

The datasets used and/or analysed during the current study are available from the corresponding author on reasonable request.

## Abbreviations

HCP: Healthcare provider
ECHO: Extension for Community Healthcare Outcomes
REDCap: Research Electronic Data Capture
