# Supplementary material for: Pediatric Project ECHO® for Pain: implementation and mixed methods evaluation of a virtual medical education program to support interprofessional pain management in children and youth
Source: BMC Med Educ. 2023 Jan 28;23:71. doi: 10.1186/s12909-023-04023-8 (PMC9883812; doi:10.1186/s12909-023-04023-8)
Supplement: Supplementary file 1 — Additional file 1. Curriculum summary of Pediatric Project ECHO for Pain (2017-2022). [file 12909_2023_4023_MOESM1_ESM.docx]

Additional file 1: Curriculum summary of *Pediatric Project ECHO for Pain* (2017-2022).

| **Program Offering** | **Dates** | **Example Topics** |
| --- | --- | --- |
| ***TeleECHO Cycle 1*** | October 2017 to June 2018 | Overview of Pain, Acute Pain Management, Acute to Chronic Pain, Opioids, Sleep, Cannabis, Neuropathic Pain, Abdominal Pain, Mind/Body Pain Management, Pain and Mental Health, Pediatric to Adult Care Transitions |
| ***TeleECHO Cycle 2*** | December 2018 to March 2019 | Pain Science, Functional Rehabilitation, Overview of Pain, Acute Pain Management, Acute to Chronic Pain, Opioids, Sleep, Cannabis, Neuropathic Pain, Abdominal Pain, Mind/Body Pain Management, Pain and Mental Health, Pediatric to Adult Care Transition, Acceptance and Commitment Therapy |
| ***TeleECHO Cycle 3*** | November 2019 to February 2020 | Pain Science, Functional Rehabilitation, Overview of Pain, Acute Pain Management, Patient Engagement, Headache, Acute to Chronic Pain, Opioids, Sleep, Cannabis, Neuropathic Pain, Abdominal Pain, Mind/Body Pain Management, Pain and Mental Health, Pediatric to Adult Care Transition, Acceptance and Commitment Therapy |
| ***TeleECHO Cycle 4*** | September 2020 to March 2021 | Overview of Pain, Speaking to children/families about pain, Virtual care for chronic pain, Pain assessment (psychological and physical), Abdominal Pain, Neuropathic Pain, Headache, Sickle Cell Pain, Pain and Medical Complexity, Procedural Pain, Pharmacological Acute Pain, Acute to Chronic Pain, Current Pain Research, Mind/Body Techniques, Physical Treatments for Pain, Cannabis, Opioids, Mental Health and Pain, Integrative Medicine and Pain, Cultural Safety, Pain and Palliative Care, Child Life Interventions for Procedural Pain, Motivational Interviewing for Pain |
| ***TeleECHO Cycle 5*** | September 2021 to March 2022 | Overview of Pain, Pain Assessment, Pain Science and Explaining Pain, Motivational Interviewing, Physical Techniques for Pain Treatment, Cultural Safety, Sickle Cell Pain, Neuropathic Pain, Hypermobility, Procedural Pain, Vaccine Pain, Acute to Chronic Pain, Child Life Interventions for Procedural Pain, Cannabis, Virtual Reality, Opioids, Medical Complexity and Pain, Pediatric to Adult Transition, Mind/Body Techniques, Interventional Pain Management |
| ***Core Competencies Cycle 1*** | November 2017 to March 2018 | Pain Science, Family Role in Pain Management, Functional Rehabilitation, Neuropathic Pain and Complex Regional Pain Syndrome, Sleep, Acceptance and Commitment Therapy, Abdominal Pain, Chronic Widespread Pain |
| ***Core Competencies Cycle 2*** | October 2018 to January 2019 | Pain Science, Pain Assessment and Diagnosis, Motivational Interviewing, Pharmacology for Pain, Physical Strategies for Pain, Cannabis, Psychological Strategies for Pain, Chronic Widespread Pain |
